# The Study of Incidence and Characteristics of Patients with Eye-Related Chief Complaints at the Emergency Department of Thammasat University Hospital

**DOI:** 10.1155/2020/4280543

**Published:** 2020-10-19

**Authors:** Intanon Imsuwan, Kumpol Amnuaypattanapon, Sakchai Vongkittirux, Yutthaphong Imsuwan

**Affiliations:** ^1^Department of Emergency Medicine, Thammasat University, Pathumthani 12120, Thailand; ^2^Department of Ophthalmology, Thammasat University, Pathumthani 12120, Thailand; ^3^Department of Ophthalmology, Phramongkutklao College of Medicine, Bangkok 10400, Thailand

## Abstract

**Background:**

Patients with eye-related chief complaints could be diagnosed not only with eye diseases but also with noneye diseases. This study determines rates and characteristics of patients with eye-related chief complaints at the Emergency Department of Thammasat University Hospital.

**Methods:**

The study design is a descriptive retrospective observational study of patients with eye-related chief complaints at the Emergency Department of Thammasat University Hospital in 2017. Demographic data, diagnosis, management, consultation, and disposition were recorded by chart review. Categorical data were reported by percentage.

**Results:**

Of the 52081 patients, 704 (1.3%) presented with eye problems. 60% of the patients were males. Patients were classified into three groups which are traumatic eye disease, nontraumatic eye disease, and noneye disease. 75.9% of the patients suffered traumatic injuries. The most common diagnoses of the traumatic eye injuries were foreign bodies at the cornea and conjunctiva and minor trauma to the conjunctiva. The most common mechanisms were foreign bodies in the eyes, cuts, or pierces. The most common causes of the injuries were from metals and housewares. The most common nontraumatic eye diagnoses were conjunctivitis and corneal ulcer. The most common noneye diagnoses were exposure of healthcare providers to secretions from patients, angioedema, and hypertensive crisis.

**Conclusions:**

Most of the patients who came to the ER with chief complaints of the eyes could be treated by doctors in the emergency room without consulting ophthalmologists. Chief complaints of the eyes could be the leading symptoms of many organ systems. Emergency physicians should be differentially diagnosed to cover neurologic, cardiovascular, and immunologic problems.

## 1. Introduction

Eye emergency conditions presented at emergency rooms include traumatic and nontraumatic eye diseases. The WHO reported that every year, 19 million patients suffer from low vision or blindness, 1.6 million patients suffer blindness from eye trauma, and 0.75 million cases require hospitalization [1]. Several studies have shown that ocular trauma cases resulting in blindness or visual impairment are preventable [2–4].

In the US, 2.5 million eye injuries occur annually; hospital charges alone amount to $200 million [5]. The total cost exceeds $5 billion including direct and indirect costs [6]. The conditions result in economical loss from medical costs and loss of working opportunities [7, 8].

The epidemiology of eye emergency has been well described in developed countries such as the US [9, 10], Europe [11], and Australia [12–14]. In the USA, 2.3 million patients (1.3%) present to emergency departments with eye problems every year [10]. The cumulative lifetime prevalence in the US of an eye injury was estimated at over 1,400 per 100,000 people [15]. In Thailand, recent data on eye emergencies are limited only to children [16].

Diagnostic errors can affect adverse patient outcomes and economic consequences, as well as account for medical liability [17, 18]. The WHO recently prioritized patient safety and included diagnostic errors as a high-priority problem. Improving diagnostic reasoning by promoting knowledge acquisition and differential diagnosis in areas at high risk of errors is one of its potential interventions [19]. Most epidemiological research uses diagnosis based on the International Classification of Diseases (ICD) system as the final diagnosis [20]. Some patients presenting with eye-related chief complaints might be diagnosed with nonophthalmic diseases. Few studies have determined groups of possible diagnoses of patients in emergency departments presenting with eye-related chief complaints. The authors are interested in groups of possible diagnoses according to chief complaints of eye problems by patients in emergency departments. The purpose of this study was to determine incidence and characteristics of patients with eye-related chief complaints at the Emergency Department of Thammasat University Hospital.

## 2. Materials and Methods

This study was performed in the Emergency Department of Thammasat University Hospital, a 750-bed inpatient tertiary-care university hospital with more than 45,000 ED visits per year. The study design involves a retrospective observational study of patients with eye-related chief complaints from January 1, 2017, to December 31, 2017. Patients with multiple chief complaints were excluded. Using the hospital information system, all records of patients with eye-related chief complaints were retrieved by using the keyword “eye.” Demographic data include patient age, gender, triage level, type of illness, and ER work shift. Outcomes include chief complaints and diagnosis. Management includes length of stay, surgery, and consultation. Outcome of management includes visual acuity at the emergency department and on follow-up visits to the ophthalmologic clinic in accordance with the International Statistical Classification of Diseases [21]. Dispositions were collected by using standard case records forms. Additional data regarding mechanism and object of injury were collected from traumatic eye patients. Injuries to the eyelid and adnexa were accorded special diagnoses. All traumatic eye injuries were classified by the Birmingham Eye Trauma Terminology (BETT) system [22].

All traumatic and nontraumatic eye-related chief complaints were included in this study. We classified patients into three groups which are traumatic eye disease, nontraumatic eye disease, and noneye disease.

This study was approved by the institutional ethical committee. All data were performed using SPSS software version 18. Numerical variables with normally distributed data were presented as the mean (SD), while non-normally distributed data were presented as the median (IQR). Categorical variables were expressed as the number and percentage.

## 3. Results

**Figure 1:**
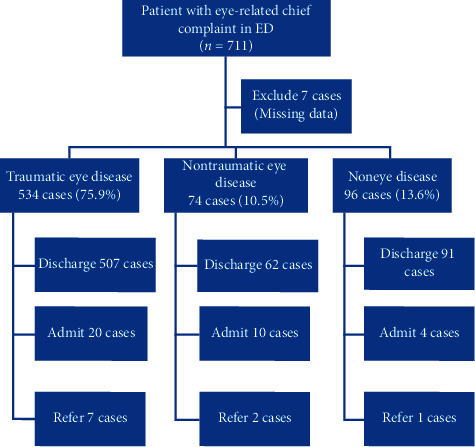
Flowchart of the study.

There were 52,081 patient visits to the ED of Thammasat University Hospital from January 1, 2017, to December 31, 2017. There were a total of 704 (1.3%) patients who had eye-related chief complaints.  Figure 1 is flow chart of the study. Characteristics of the patients in the study are presented in Table 1.

Most of the patients were males. The most common age group in this study was 21–30 years. Most of the patients were traumatic patients and classified in the urgency triage level. There was one patient in the emergency triage level whose chief complaint was swollen eyes with anaphylactic shock. The most common chief complaints were eye pain (79.8%), followed by swollen eye (7.8%) and bruise at the eye (3.3%). We classified patients into three groups, which are traumatic eye disease patients, 534 (75.9%), nontraumatic eye disease patients, 74 (10.5%), and noneye disease patients, 96 (13.6%).

For the group of traumatic eye disease, the most common diagnoses were foreign body at the cornea and conjunctiva (30.1%), minor trauma to conjunctiva (20%), and chemical injury (11.6%). For the group of nontraumatic eye disease, the most common diagnoses were conjunctivitis (20.3%), corneal ulcer (20.3%), and lid inflammation (13.5%). The most common diagnoses for the noneye disease group were healthcare-associated exposures affecting medical professionals (37.5%), angioedema (35.4%), and hypertensive urgency (5.2%), as shown in Table 2.

In the trauma group, the most common mechanisms of injury were foreign body (57.4%), cuts or pierces (11.1%), and animal bite or sting (6.7%). The most common objects were metal (22.1%), household products (14.1%), and dust (11.7%), as shown in Table 3.

Eye pain is the most predominant chief complaint in traumatic eye disease followed by a bruise at the eye and red eye. Foreign bodies at the cornea or conjunctiva must be extracted by emergency physicians. Bruises at the eyes are associated with blunt traumatic eye injuries. Patient with serious eye vision complaints are likely to suffer penetrating eye injuries or traumatic optic neuropathy. We classified diagnoses of traumatic eye diseases by chief complaints, as shown in Table 4.

Eye pain is the most common chief complaint in nontraumatic eye disease patients followed by swollen and itching eye. Most of the diagnoses were eye-related problems. But, we found some noneye problems presented with eye pain. Some patients with eye pain, fever, and swollen eyes were diagnosed with cavernous sinus thrombosis. Emergency physicians should be aware of noneye problems in patients with eye complaints with systemic symptoms. Swollen and itching eye are common complaints for allergic conjunctivitis and lid inflammation; for example, hordeolum is a nonemergency eye problem. Another serious diagnosis that should be recognized is orbital cellulitis, which is usually presented with swollen eye as well. We also found nonemergency problems in the emergency department; for example, cataract presented with blurred vision. Patients are often concerned about red eye. Red eye which is clearly seen in white sclera is mostly diagnosed as subconjunctival hemorrhage, which does not impact vision, whereas red eye which is seen only surrounding the limbus is a true emergency condition, such as corneal ulcer or endophthalmitis. Therefore, emergency physicians should be concerned about serious conditions that could be presented with common chief complaints. We classified diagnoses of nontraumatic eye diseases by chief complaints, as shown in Table 5.

We summarized eye-related chief complaints related to organ systems in Table 6. Swollen eyes are probably caused by allergy or animal bite or sting. Vision loss and double vision are both symptoms related to the neurologic system. Vision loss could be a symptom of stroke which is a time-sensitive condition. Double vision might be diagnosed as thalamic infarction or strabismus. Blurred vision is related to high blood pressure which is possibly an emergency condition such as hypertensive emergency or even hyperglycemia due to transient diabetic hyperopia [23]. Broad diagnosis should be considered by emergency doctors for preventing diagnostic error and maintaining quality assurance [24–26].

In our study, we found no significant difference in diagnosis between emergency physicians and ophthalmologists. But, interestingly, we found some conditions in traumatic patients in which patients should have consulted ophthalmologists for appropriate management. Patients who were injured with high-velocity objects to the eye, especially occupation-related eye injuries (for example, blower fan, nail, wire, and metal chip), are at high risk of serious eye injuries. We found delayed hyphema on follow-up visits after only small findings such as conjunctivitis had been diagnosed at the emergency room. It is probable that minimal hyphema cannot be seen on initial assessment. Chemical injury is one of the true eye emergency conditions that need ophthalmologic consultation. The rationale for consultation is that the severity of corneal injury cannot be fully evaluated without a slit lamp. The management and outcome would be better if patients had proper initial management. Any traumatic subconjunctival hemorrhage is another condition that should be referred to ophthalmologists. There might be penetrating injury to the globe that needs emergency management. We recommend immediate ophthalmologic consultation in high mechanism injury. The final diagnosis of patients who were discharged by emergency physicians and, then, consulted ophthalmologists is shown in Table 7.

**Figure 2:**
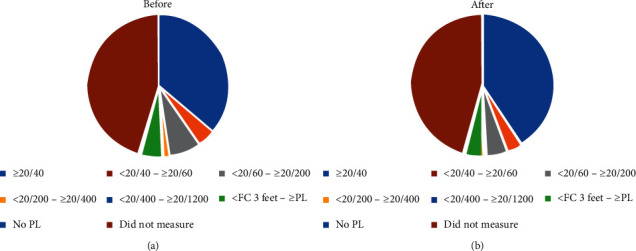
Visual acuity before and after management.

Most of the management involved eye irrigation, topical eye drops, and removal of foreign bodies. Most of the patients could be managed by doctors in the emergency room and discharged. Outcomes of patients including management, surgery, consultation, and disposition are shown as Table 8. Visual acuity before and after management are shown in Figure 2.

Most of the patients (79.5%) were discharged without consultation. Almost 19% of patients consulted an ophthalmologist at the emergency room for appropriate management. The other specialties which were consulted were medicine, pediatrics, surgery, and obstetric-gynecology, according to the patient condition and indication for consultation. Patients who had consultation stayed in the emergency department longer than those who were discharged by an emergency doctor. Emergency department length of stay classified by consultation is shown in Table 9.

## 4. Discussion

Emergency physicians generally encounter emergency patients with symptom-oriented complaints. The current study is the first report of emergencies in which eye-related symptoms are the primary chief complaint. These can include minor symptoms in which the eye itself is the problem or critical problems from other systems affecting the eye.

From the data presented, it can be seen, not surprisingly, that eye pain is the most common eye-related chief complaint of emergency patients. There are several symptoms similar to eye pain; for example, burning eye, throbbing at eye, and feeling something in eye. Moreover, patients sometimes explain their symptoms as eye pain even though they have more than one symptom, for example, red eye as well. The most common chief complaint in our study was eye pain which can be varied on diagnosis. On the other hand, blurred vision and vision loss were less common but seem to more likely be serious eye conditions; for example, central retinal artery occlusion (CRAO) should be considered high risk [27]. Swollen eyes should be recognized as possible of anaphylaxis which is a life-threatening hypersensitivity reaction and should be treated as an emergency [24].

In the triage system of our study, authors found only one emergency patient whose chief complaint was swollen eyes with shock after taking acetaminophen and was finally diagnosed as anaphylactic shock. Since all clinical symptoms are compatible with anaphylaxis, intramuscular epinephrine injection treatment was the first priority for this patient. It is rather unlikely that acetaminophen is a cause of anaphylaxis because we know that NSAIDs and beta lactam are the most common drug-induced anaphylaxis [28, 29]. Previous studies reported drugs combined with acetaminophen as a cause of anaphylaxis [29]. The explanation for this result can be that some conditions mimic anaphylaxis [30, 31], but definite diagnosis and further information about this patient is limited due the retrospective nature of the study.

Nevertheless, we found undertriage in true eye emergency conditions in which patients were triaged as urgent, for example, central retinal artery occlusion and chemical eye injury. A true eye emergency condition which is a chief complaint associated with some types of visual loss; for example, blurred vision or visual loss should be triaged as ESI level 2 [27]. The reason is the triage nurse needs to have some experience for screening patients, and measuring visual acuity cannot be practical for emergency nurses for all patients with eye-related symptoms because some eye emergency conditions need emergency management. Some limitations may be patient concern. Previous studies have shown some triage screening tools for eye emergency conditions [32]. Training triage nurses about emergency ocular conditions as high-risk situations can improve triage management of emergency eye conditions [33, 34].

Our findings were similar to those reported in previous studies, showing that the largest group of patients is middle-aged males [9, 10, 35]. The reason for those results is that adult males have a higher tendency for risk-taking behavior and a higher proportion of occupation-related eye injuries.

Traumatic patients are the largest group of our study which is similar to the previous literature [9, 20]. Our findings showed that foreign body at the cornea and conjunctiva are the most common diagnoses in this group. Channa reported corneal abrasion as the most common diagnosis in the emergency category, followed by a corneal foreign body. Varizi reported corneal injury without a foreign body and corneal foreign body [9, 20]. Corneal abrasion typically resulting from mechanical injuries is often associated with foreign bodies. The reason for the cause and mechanism of corneal abrasion by foreign body or foreign body at cornea are similar; therefore, the diagnosis would be the same. Previous studies diagnosed corneal abrasion by using slit lamps. In our study, the diagnosis of corneal abrasion would have been higher if we had used the accuracy of a slit lamp for diagnosis.

Similar to a previous report [10], foreign bodies were the most common mechanism of injury in our study. The exact location is undefined due to the retrospective nature of this study. But, we found many objects related to occupation-related eye injuries. Metal was the most common object followed by chemical products, welding, and construction materials. From the previous studies, chips, particles, and chemicals were the main sources of work-related eye injuries. Foreign bodies and chemicals caused more than two-thirds of these injuries [36].

The current study found fewer nonemergency patients and nontraumatic patients than the previous studies [9, 10], probably due to the present emergency triage system which screens nonemergency patients to the eye clinic during regular hours. Patients mostly come in morning and afternoon shifts. The reason is probably due to various factors; for example, the occupation-related eye injury patients are more likely to come in daytime and traumatic injury is the largest group in our study [37]. The most common diagnosis in nonemergency patients was conjunctivitis, similar to the previous studies [9, 20].

Authors found that about 14% of patients with eye-related chief complaints were diagnosed with noneye diseases. The management of noneye diseases is different from that of general eye conditions and should be managed by a doctor who works in an emergency room. It means that patients with high-risk conditions should be triaged by the current emergency system before sending them to an ophthalmology clinic. Besides, the most common diagnosis in this group, which is healthcare-associated exposure affecting medical professionals, requires coming to the emergency department. Severe allergic reaction and hypertensive crisis are the two common groups which need to be treated in emergency rooms.

Healthcare-associated exposures affecting medical professionals were the most common cause of noneye disease resulting in eye-related chief complaints. The authors found eye-related exposures due to various medical procedures by healthcare providers including both physicians and nurse practitioners. Most of the exposures were low-risk events; for example, intravenous fluid or medicine splash to the eyes. Hospital regulations or hospital policies should encourage wearing protective eye glasses while performing medical procedures [38].

Angioedema, which is likely presented with swollen eyes, was the second most common cause of eye-related complaints regarding noneye diseases. Our study found that seafood and NSAIDs were the most common causes of angioedema. Doctors in emergency rooms should look for all possible allergens and follow-up with an allergist for further investigation and management.

The common management of patients includes emergency procedures; for example, eye irrigation and foreign body removal. The emergency doctor should be proficient in these techniques and know limitations before consultation.

Globe rupture and penetrating eye injuries are common diagnoses requiring admission and surgery. Our results are similar to the previous studies in developing countries due to epidemiological pattern and high incidence of occupation-related eye injuries [35]. On the other hand, those results are different from the previous studies in developed countries due to slightly different inclusion criteria [20].

Most of the patients were managed by emergency doctors without ophthalmologic consultation. The most common diagnoses for traumatic groups which required ophthalmologic consultation were foreign body at the cornea, penetrating eye injuries, and corneal abrasion. For the nontraumatic groups, they were corneal ulcer and acute angle closure glaucoma. These generally required appropriate consultation from standard management [39]. But, some conditions, such as foreign body at the cornea and corneal abrasion, remained unclear due to various factors depending on institution and availability of slit lamps in emergency rooms and ophthalmologists. In our settings, as a university hospital, consultation was acceptable if the emergency doctor was concerned about a high-risk condition and lacked a slit lamp in the emergency room. Another reason for consultation might be that emergency doctors do not feel well-trained or they are uncomfortable performing eye examinations [40]. Defining specific objectives for using slit lamps to diagnose emergency eye conditions in ophthalmologic rotation of emergency resident training programs could improve confidence and quality of emergency care [41].

According to Table 9, the length of stay of patients who were discharged by emergency doctors was less than that of patients who had consultations. There is no doubt that more throughput and output process in major specialties increase the duration of stay [42]. The reason that the length of stay at ophthalmology consultation was shorter than that at other specialty departments is that most of the patients are ambulate and can move to the ophthalmologic clinic to use a slit lamp and discharge after consultation.

A limitation of our study is lack of data due to our retrospective methodology. As a single study within a tertiary-care setting, generalizability is limited beyond this context. Additionally, we used diagnoses by doctors who worked in the emergency department, but some injuries have more than one diagnosis by ophthalmologists. We used the most severe diagnosis for the primary diagnosis. We generally captured the primary reason for the emergency department visit as the eye-related chief complaint which is inclusion criteria slightly different from the previous studies, but it is possible that, in some cases, noneye chief complaints were finally diagnosed as eye emergency conditions; for example, punch to the face might cause orbital floor fracture. The associated injury from any trauma which is likely to be diagnosed from ICD10 might not be presented as a chief complaint. Those reasons are some of the factors that confound our results. Future research should be multicenter and multiyear to investigate the influence of variability of settings.

## 5. Conclusions

The most common chief complaint about eyes were eye pain followed by swollen eyes and bruise at eyes. Most of the patients were from trauma. The most common diagnoses of the traumatic eyes were foreign bodies at the cornea and conjunctiva, minor trauma to the conjunctiva, and chemical injuries. The most common mechanisms were foreign bodies in the eyes, cut or pierce, and animals or insect attacks. The most common causes of the injuries were from metals, housewares, and dust. The most common nontraumatic eye diagnoses were conjunctivitis, corneal ulcer, and lid inflammation. The most common noneye diagnoses were healthcare-associated exposures affecting medical professionals, angioedema, and hypertensive crisis.

Most of the patients who came to the ER with chief complaints of the eyes could be treated by doctors in the emergency room without consulting the ophthalmologists. Chief complaints of the eyes could be the leading symptoms of many organ systems. The emergency physicians should differential diagnose to cover cardiovascular and allergic problems.

## Figures and Tables

**Table 1:** Characteristics of 704 patients with eye-related chief complaints.

Variable	Total number (%) (*n* = 704)
Male gender	421 (59.8)
Age, year†	31 (20, 41)
Age group, year	
<18	111 (15.6)
18–44	438 (61.4)
45–64	114 (16)
≥65	50 (7)
Triage level	
Immediate	—
Emergency	1 (0.1)
Urgent	677 (96.2)
Nonurgent	26 (3.7)
Type of illness	
Trauma	596 (84.7)
Nontrauma	108 (15.3)
ED shift	
Morning	304 (43.2)
Afternoon	292 (41.5)
Night	108 (15.3)
Chief complaint	
Eye pain	562 (79.8)
Swollen eye	55 (7.8)
Bruise at the eye	23 (3.3)
Blurred vision	21 (3.0)
Red eye	18 (2.6)
Itching eye	13 (1.8)
Vision loss	5 (0.7)
Bleeding from the eye	4 (0.6)
Double vision	2 (0.3)
Cannot close the eyelids	1 (0.1)
Diagnosis	
Traumatic eye disease	534 (75.9)
Nontraumatic eye disease	74 (10.5)
Noneye disease	96 (13.6)

†Median (interquartile ranges); ED: emergency department.

**Table 2:** Proportions of patients with eye-related chief complaints classified by groups of diagnoses.

Variable	Total (*n* = 704)
Traumatic eye disease	534
Foreign body at the cornea and conjunctiva	161 (30.1)
Minor trauma to the conjunctiva	107 (20.0)
Chemical injury	62 (11.6)
Eyelid injury	39 (7.3)
Corneal abrasion	37 (6.9)
Animal bite/sting	31 (5.8)
Subconjunctival hemorrhage	26 (4.9)
Ultraviolet keratitis	22 (4.1)
Penetrating eye injuries	13 (2.4)
Hyphema	10 (1.9)
Globe rupture	9 (1.7)
Vitreous hemorrhage	4 (0.7)
Lacrimal passage and apparatus injury	3 (0.6)
Macular problem in close globe injury	3 (0.6)
Intraocular FB (IOFB)	2 (0.4)
Lens subluxation	2 (0.4)
Traumatic mydriasis	2 (0.4)
Traumatic optic neuropathy	1 (0.2)

Nontraumatic eye disease	74
Conjunctivitis	15 (20.3)
Corneal ulcer	15 (20.3)
Lid inflammation	10 (13.5)
Subconjunctival hemorrhage	6 (8.1)
Acute glaucoma	5 (6.8)
Preseptal cellulitis	4 (5.4)
Endophthalmitis	3 (4.1)
Uveitis	2 (2.7)
Cavernous sinus thrombosis	2 (2.7)
Cataract	2 (2.7)
Strabismus	2 (2.7)
CRAO	1 (1.4)
CRVO	1 (1.4)
Orbital cellulitis	1 (1.4)
Herpes zoster ophthalmicus	1 (1.4)
Pinguecula	1 (1.4)
Retinal tear/detachment	1 (1.4)
Floater	1 (1.4)
Postophthalmic surgical complication	1 (1.4)

Noneye disease	96
Healthcare-associated exposure	36 (37.5)
Angioedema	34 (35.4)
Hypertensive urgency	5 (5.2)
Anaphylaxis	4 (4.2)
Hypertensive emergency	3 (3.1)
Acute kidney injury	3 (3.1)
Thalamic infarction	1 (1)
Preeclampsia	1 (1)
Bell's palsy	1 (1)
Tension headache	1 (1)
Henoch–Schonlein purpura	1 (1)
Hyperglycemia	1 (1)
Amaurosis fugax	1 (1)
Miscellaneous	4 (4.2)

**Table 3:** Characteristics of traumatic eye patients.

Variable	Total (*n* = 596)
Mechanism of injury	
Foreign body	342 (57.4)
Cut or pierce	66 (11.1)
Animal bite or sting	40 (6.7)
Blunt trauma	36 (6.0)
Medical treatment	34 (5.7)
Ultraviolet light	22 (3.7)
Physical assault	18 (3.0)
Sport-related	14 (2.3)
Falling	11 (1.8)
Motor vehicle	10 (1.7)
Fire	3 (0.5)

Object	
Metal	132 (22.1)
Household products	84 (14.1)
Dust	70 (11.7)
Chemical products	69 (11.6)
Human body parts	57 (9.6)
Animal	40 (6.7)
Wood	25 (4.2)
Welding	22 (3.7)
Contact lens	19 (3.2)
Construction	18 (3.0)
Food	13 (2.2)
Stone	9 (1.5)
Medicine	8 (1.3)
Plastic	7 (1.2)
Gardening	7 (1.2)
Fire	3 (0.5)
Unknown	13 (2.2)

**Table 4:** Diagnosis of traumatic eye disease classified by the chief complaint.

Chief complaint	Diagnosis
Eye pain (90%)	Foreign body at the cornea or conjunctiva, minor trauma to the conjunctiva, and chemical injury
Bruise at the eye (4%)	Eyelid injury, subconjunctival hemorrhage, corneal abrasion, traumatic mydriasis, globe rupture, and lens subluxation
Red eye (2%)	Minor trauma to the conjunctiva, corneal abrasion, hyphema, and subconjunctival hemorrhage
Swollen eye (1%)	Eyelid injury, animal bite, or sting
Blurred vision (1%)	Penetrating eye injuries and vitreous hemorrhage
Bleeding from eye (<1%)	Hyphema and animal bite
Vision loss (<1%)	Traumatic optic neuropathy

**Table 5:** Diagnosis of nontraumatic eye disease classified by the chief complaint.

Chief complaint	Diagnosis
Eye pain (55%)	Corneal ulcer, lid inflammation, acute glaucoma, conjunctivitis, preseptal cellulitis, uveitis, endophthalmitis, and cavernous sinus thrombosis
Swollen eye (14%)	Allergic conjunctivitis, lid inflammation, orbital cellulitis, and preseptal cellulitis
Itching eye (9%)	Allergic conjunctivitis and lid inflammation
Red eye (8%)	Subconjunctival hemorrhage, endophthalmitis, corneal ulcer, and conjunctivitis
Blurred vision (5%)	Endophthalmitis, corneal ulcer, and cataract
Vision loss (4%)	Acute glaucoma, central retinal artery occlusion, and central retinal vein occlusion
Bleeding in the eye (3%)	Lid inflammation and postophthalmic surgical complication
Double vision (1%)	Strabismus

**Table 6:** Characteristics of noneye disease classified by the group of diagnoses.

Chief complaint	Organ system	Diagnosis
Eye pain	Neurology	Healthcare-associated exposure, animal bite or sting, and tension headache
Swollen eye	Immunology	Angioedema, anaphylaxis, animal bite or sting, and Henoch–Schonlein purpura
Blurred vision	Cardiovascular and endocrinology	Hypertensive emergency, hypertensive urgency, severe preeclampsia, and hyperglycemia
Vision loss	Neurology	Amaurosis fugax
Double vision	Neurology	Thalamic infarction
Cannot close the eyelids	Neurology	Bell's palsy

**Table 7:** Comparison of final diagnosis of patients who were discharged by emergency physicians and consulted ophthalmologists.

Mechanism of injury	Diagnosis by an emergency physician	Diagnosis by an ophthalmologist
*Patients who were discharged by emergency physicians*		
Fan blade struck the eye	Conjunctivitis	Subconjunctival hemorrhage
Nail hit the eye	Minor trauma to the conjunctiva	Delayed hyphema
Water balm paste the eye	Chemical injury	Corneal abrasion
Plastic box scratched the eye	Subconjunctival hemorrhage	Conjunctival laceration
Wire hit the eye	Subconjunctival hemorrhage	Conjunctival laceration

*Patients who were consulted by emergency physicians*		
Air gun splashed the eye with water	Minor trauma to the conjunctiva	Chemosis with periorbital subcutaneous emphysema
Scrap metal hit the eye	Minor trauma to the conjunctiva	Subconjunctival metallic foreign body with partial thickness scleral laceration
Burning paper burned the eye	Corneal abrasion	Corneal abrasion with corneal ulcer
Fan blade burst and hit the eye	Hyphema	Traumatic hyphema with tear bulbar conjunctiva and commotio retinae with a traumatic macula hole
Fiber scratched the eye	Hyphema	Traumatic hyphema with laceration in the upper eyelid and traumatic iridodialysis
Air hose splashed the eye	Hyphema	Traumatic hyphema with subconjunctival hemorrhage

**Table 8:** Management and outcome of 704 eye-related chief complaint patients.

Variable	Total (*n* = 704)
Management	
Eye irrigation	265 (37.6)
Topical antibiotic eye drop	118 (16.8)
Observation	114 (16.2)
Remove foreign body	106 (15.1)
Radiograph	39 (5.5)
IV antihistamine	38 (5.4)
Anesthetic eye drop	9 (1.3)
Human rabies immunoglobulin	5 (0.7)
Suture	5 (0.7)
Oral antihypertensive drug	1 (0.1)
IV antihypertensive agent	1 (0.1)
Blood transfusion	1 (0.1)
IV antibiotics	1 (0.1)
IV analgesia	1 (0.1)
Surgery	
Yes	26 (3.7)
No	678 (96.3)
Consultation	
Ophthalmologist	133 (18.9)
Medicine	7 (1)
Pediatrics	2 (0.3)
Surgery	1 (0.1)
OB-GYN	1 (0.1)
No	560 (79.6)
Disposition	
Discharge	
(i) ER discharge	225 (32)
(ii) Follow-up with ophthalmology clinic	311 (44.2)
(iii) Follow-up with other specialty clinic	31 (4.4)
(iv) Consult ophthalmologist and discharge	93 (13.2)
Admit	
(i) Ophthalmology	30 (4.3)
(ii) Medicine	3 (0.4)
(iii) OB-GYN	1 (0.1)
Refer	10 (1.4)

**Table 9:** Emergency department length of stay classified by consultation.

Variable	Number	Length of stay; median (IQR)
No consultation	560	87 (34, 103)
Consultation		
Ophthalmologist	133	160 (72, 199)
Medicine	7	269 (110, 438)
Pediatrics	2	224 (115)
Surgery	1	322
OB-GYN	1	105
Total	704	103

## Data Availability

The data of this study are available from the corresponding author upon request.
